# Assessing Physical Activities Occurring on Sidewalks and Streets: Protocol for a Cross-Sectional Study

**DOI:** 10.2196/12976

**Published:** 2019-07-30

**Authors:** Richard Robert Suminski Jr, Gregory Dominick, Philip Saponaro

**Affiliations:** 1 Center for Innovative Health Research Department of Behavioral Health and Nutrition University of Delaware Newark, DE United States

**Keywords:** observation, machine learning, behavior, environment, public health

## Abstract

**Background:**

A considerable proportion of outdoor physical activity (PA) is done on sidewalks and streets, necessitating the development of a reliable measure of PA performed in these settings. The Block Walk Method (BWM) is one of the more common approaches for this purpose. Although it utilizes reliable observation techniques and displays criterion validity, it remains relatively unchanged since its introduction in 2006. It is a nontechnical, labor-intensive, first generation method. Advancing the BWM would contribute significantly to our understanding of PA behavior.

**Objective:**

This study will develop and test a new BWM that utilizes a wearable video device (WVD) and computer video analysis to assess PAs performed on sidewalks and streets. The specific aims are to improve the BWM by incorporating a WVD (eyeglasses with a high-definition video camera in the frame) into the methodology and advance this WVD-enhanced BWM by applying machine learning and recognition software to automatically extract information on PAs occurring on the sidewalks and streets from the videos.

**Methods:**

Trained observers (1 wearing and 1 not wearing the WVD) will walk together at a set pace along predetermined 1000 ft sidewalk and street observation routes representing low, medium, and high walkable areas. During the walks, the non-WVD observer will use the traditional BWM to record the numbers of individuals standing, sitting, walking, biking, and running in observation fields along the routes. The WVD observer will continuously video the observation fields. Later, 2 investigators will view the videos to determine the number of individuals performing PAs in the observation fields. The video data will then be analyzed automatically using multiple deep convolutional neural networks (CNNs) to determine the number of humans in the observation fields and the type of PAs performed. Bland Altman methods and intraclass correlation coefficients (ICCs) will be used to assess agreement. Potential sources of error such as occlusions (eg, trees) will be assessed using moderator analyses.

**Results:**

Outcomes from this study are pending; however, preliminary studies supporting the research protocol indicate that the BWM is reliable for determining the PA mode (Cramer V=.89; *P*<.001), the address where the PA occurred (Cohen kappa*=*.85; *P*<.001), and the number engaged in an observed PA (ICC=.85; *P*<.001). The number of individuals seen walking along routes was correlated with several environmental characteristics such as sidewalk quality (*r*=.39; *P*=.02) and neighborhood aesthetics (*r*=.49; *P*<.001). Furthermore, we have used CNNs to detect cars, bikes, and pedestrians as well as individuals using park facilities.

**Conclusions:**

We expect the new approach will enhance measurement accuracy while reducing the burden of data collection. In the future, the capabilities of the WVD-CNN system will be expanded to allow for the determination of other characteristics captured in videos such as caloric expenditure and environmental conditions.

**International Registered Report Identifier (IRRID):**

PRR1-10.2196/12976

## Introduction

### Background

Physical inactivity facilitates the development of chronic diseases including obesity, cardiovascular disease, type 2 diabetes, and some cancers and independently contributes to nearly 11% of total annual US health care expenditures [1-4]. Despite national and local efforts to increase physical activity (PA), approximately 51% of US adults are not sufficiently active to achieve health benefits and less than 55% of children younger than 19 years engage in an hour of PA or more per day [3,4]. Whereas psychosocial factors associated with PA are traditionally targeted within behavioral interventions, such approaches have had limited impact on population-level behavior change unless integrated into more comprehensive, multilevel (eg, individual and community) efforts [5]. Indeed, the evidence has grown exponentially regarding the key role contextual influences of the social and physical environments existing within communities play in shaping health behaviors such as PA [6-9]. As such, community-level interventions targeting environmental factors and policies affecting these factors are highly recommended and are becoming the *approach of choice* for promoting PA [6,10].

Neighborhood built environment characteristics have been studied extensively over the past 10 years and are some of the strongest correlates of PA [9,11]. For instance, higher levels of pedestrian PA have been linked to mixed-land use, access to destinations, and street/sidewalk connectivity [12-14]. Moreover, some built environment characteristics are shown to influence the degree to which individuals engage in PA [15-17]. In particular, access to neighborhood sidewalks and streets is associated with greater participation in moderate-to-vigorous PA [18-20]. Sidewalks and streets are among the most common aspects of the built environment where a considerable proportion of outdoor, PAs (eg, walking, running, and cycling) are performed largely within neighborhoods that are proximal to a person’s home [19,21,22]. For example, approximately 70% of adults who engage in recreational walking report using the sidewalks and streets in their neighborhood, and adults who are physically active near their homes gain about 17% more time in daily moderate-to-vigorous PA [21,22].

Studies and evaluations of PAs performed on sidewalks and streets, whether to detect changes in usage or determine how associated environmental conditions impact their usage, necessitate a reliable, accurate, and easily administered approach for assessing PA. Self-report questionnaires are hampered by recall bias, plus they have not been adequately validated for geo-locating PAs [22-24]. This is particularly true when asking respondents if they were physical active on the sidewalks and streets in their neighborhood [22,25,26]. Objective measures including accelerometers and pedometers, combined with global positioning systems (GPSs) have been used to geo-locate PAs [27,28]. Although an improvement over self-report questionnaires, drawbacks exists. First, the logistics and cost to use these in community-level evaluations is prohibitive. Second, accelerometers and pedometers provide no information on the location of the activity and even when coupled with GPSs, only the sample of individuals (cohort) wearing the monitor/GPS are counted, and their PA data are restricted to the geographical locations they visited. As with recall questionnaires, monitor/GPS are not useful for determining utilization rates of specific geographical areas such as sidewalks and streets.

In contrast, the observation method is a reliable approach to counting the number of individuals engaged in various PAs in different environmental settings [29-34]. It is especially useful for determining human usage of sidewalks and streets and widely employed by transportation departments to count pedestrians. In this context, it is referred to as a pedestrian count and involves a stationary observer who records the volume and direction of pedestrian traffic along various routes [35]. Our research team has converted the pedestrian count method to a mobile observation method called the Block Walk Method (BWM) [32,33]. It is reliable, and PAs assessed with the BWM are significantly associated with microlevel environmental characteristics (eg, sidewalk defects and crosswalks) [32-34,36].

The BWM uses time sampling techniques in which observers actually walk predefined segments of sidewalks and streets at a set pace while systematically chronicling the number of individuals performing activities of interest (eg, walking and cycling). The BWM is better than pedestrian counts because it captures a substantially greater proportion of the sidewalks and streets, and thus, a wider spectrum of environmental exposures and a richer context in which to explore PA behavior. Mobile observers, as used in the BWM, provide a very objective, precise, scientifically rigorous, and replicable way to assess PAs performed in diverse environmental conditions. Despite the BWM’s many benefits, it has not been updated since its introduction in 2006, and limitations inherent in its original design are still present. In its current form, the BWM is time consuming, requires extensive training, and has questionable accuracy when observing larger groups.

The extension of video technology within mobile and wearable video devices (WVDs) provides extraordinary opportunities for objectively measuring georeferenced imagery including sidewalk and street users in real time. It is now feasible to leverage these technologies to supplement or replace the traditional observational methods used by the BWM. Until recently, video recording devices were bulky, and the video resolutions were crude. Video recorders can now be embedded into the frame of a pair of sunglasses or attached to an unmanned aerial vehicle to provide a completely new, more robust vantage point. Although video capture has not been used to study PAs on sidewalks and streets, it has been used along with computer vision techniques to identify and classify people in different PA intensities (eg, light, moderate, and vigorous) [37,38]. However, findings are based on small study samples in which videos were recorded from stationary cameras within controlled settings. As such, the algorithms developed to predict PA intensity are not generalizable to free-living PA that occurs within open public spaces. Researchers have noted that the use of videos for research purposes is safer, less costly, more efficient, and more precise than traditional approaches [39]. The adaptation of current video technology to the study of PA behavior on sidewalks and streets is a logical next step in the evolution of PA measurement. Therefore, we propose a highly innovative study using a WVD to acquire information on sidewalk and street users. Further, we will analyze the videos automatically using a machine learning technique known as deep convolutional neural networks (CNNs). Deep CNNs have the ability to detect and classify objects in a scene. State-of-the-art CNNs such as You Only Look Once have been trained on millions of images from typical large datasets such as ImageNet and COCO to be able to recognize thousands of object types in real time [40-42]. Other CNNs have been trained on a narrower group of object types such as pedestrians only [43,44]. Typically, CNN approaches are more robust than traditional computer vision approaches and work with “in the wild” data. This robustness is because of the data-driven nature, which learns to ignore image artifacts and noise implicitly.

### Objectives

As described above, the BWM (and PA observation methods in general) has limitations. Whether today’s technology can be used to alleviate these limitations in human populations is virtually unknown. The proposed study seeks to develop and test a new BWM that utilizes a WVD and computer video analysis to assess PAs performed on sidewalks and streets. The following aims will be completed to accomplish this objective: *Aim 1:* Improve the BWM by incorporating a WVD into the methodology. The WVD is a pair of eyeglasses with a high definition video camera embedded into the frames. We expect the WVD to be a viable option for improving the acquisition and accuracy of data collected using the BWM. *Aim 2:* Advance the WVD-enhanced BWM by applying machine learning and recognition software to automatically extract information on PAs occurring on the sidewalks and streets from the videos.

## Methods

### Aim 1

#### Overview

For this cross-sectional study, we will first identify low, medium, and high walkability areas of different size cities. Afterwards, we will randomly select a sample of observation routes (1000 foot long street segments) from each walkability and city strata. The BWM will then be conducted along each observation route on 2 different days and at 6 different times. A total of 2 observers will perform the BWM simultaneously. A total of 1 observer will follow the traditional BWM procedures, whereas the other walks side-by-side with this observer and records video using the WVD. Later, 2 investigators will review the videos and, based on the BWM criteria for counting individuals, derive independent counts of individuals being physically active on sidewalks and streets. Comparative analyses will be conducted to determine the equivalence of the 2 approaches.

#### Observation Areas: Cities and Walkability

We are stratifying our sample to observe PAs occurring along sidewalks and streets given a wide range of conditions related to city size and walkability. We selected 3 cities: West Chester, Pennsylvania; Wilmington, Delaware; and Philadelphia, Pennsylvania that are small, medium, and large in terms of population, respectively (Multimedia Appendix 1) [45]. City size was considered a strata because it is associated with factors (eg, local norms, population density, and types of destinations) that could influence how humans use sidewalks and streets. In essence, our goal is to increase the generalizability of this study’s outcomes.

Drawing from our familiarity with the study cities and examinations of aerial maps, we will identify 3 neighborhoods per city we estimate as being low, medium, and high walkability. This is being done to streamline the process because there are 44, 92, and 160 defined neighborhoods in West Chester, Wilmington, and Philadelphia, respectively. Afterwards, we will actually measure walkability for each selected neighborhood using WalkScore. As WalkScores can vary across neighborhoods, we will base a neighborhood’s WalkScore on the average of WalkScores for 10 randomly selected addresses drawn from a list of all addresses in the neighborhood. This process will be repeated until 1 low (WalkScore ≤33), 1 medium (WalkScore 33 to ≤66), and 1 high (WalkScore >66) walkable neighborhood is located in each city giving us a total of 9 neighborhoods. We are using WalkScore because it is a valid measure for estimating walkability [46-49]. It is significantly correlated with geographical information system–derived indicators of neighborhood walkability such as the availability of retail destinations, intersection density, amenities, street connectivity, residential density, and access to public transit provisions [46-48]. In addition, a higher Walk Score is significantly associated with minutes/week of transport and leisure walking independent of sociodemographic and health variables [49]. WalkScore uses publicly available data from various sources (Google, Open Street Map, and Localeze) and an algorithm to assign a score to a location based on the straight-line distance to various categories of amenities (eg, schools, stores, parks, and libraries) weighted by facility type priority and a distance decay function [50]. The result is a walkability score between 0 and 100, with 0 being the least walkable and 100 being the most walkable. The location can be entered as geographic coordinates or as an address which is then geolocated using Google Geolocation [50].

#### Observation Routes

The total linear length of sidewalks and streets in the 9 neighborhoods will be estimated using the ruler tool in Google Earth (a geobrowser that accesses satellite, aerial imagery, and other geographic data to represent the Earth as a 3-dimensional globe). The ruler tool is a geographical information systems-based application with submeter resolution. We have found the ruler tool to be accurate to within ±1.5% for measuring street segment lengths. Based on our previous work, we expect an average of 180,000 total linear ft. of sidewalks and streets per neighborhood [32-34]. The total linear ft./neighborhood will be divided into 1000 ft. routes, and a sample of these routes representing 20% of the total number of routes in a neighborhood will be randomly selected for study, which is an adequate percentage to obtain a representative sample [51]. Given our expectations, this would equate to an average of 36 observation routes per neighborhood or a total of 324 observation routes.

#### Observation Schedule

Each observation route in a neighborhood will be observed 3 times on a weekday and 3 times on a weekend day, which will give us a stable estimate of the outcome variable [52]. Each observation period will last 10 min and occur during each of the following time periods: 8 to 9 am, 12 to 1 pm, and 5 to 6 pm (*note: all observations will occur during daylight hours*). We will be able to complete observations of approximately 6 to 8 observation routes per week meaning a total of 5 to 6 weeks will be needed to assess all observation routes in a given neighborhood. To reduce ordering and seasonal effects, observations will be conducted in only 1 neighborhood per day with each day randomly selected from the pool of days available for the 12-month period when the BWM will be conducted. To reduce the effects of ordering within a time period, observation routes will be numbered consecutively and then placed in a random order for the observation schedule. Observations will not be conducted on days having an event that would affect counts (eg, parade and marathon) or during times when it is raining or snowing.

#### Block Walk Method Procedures

**Figure 1:**
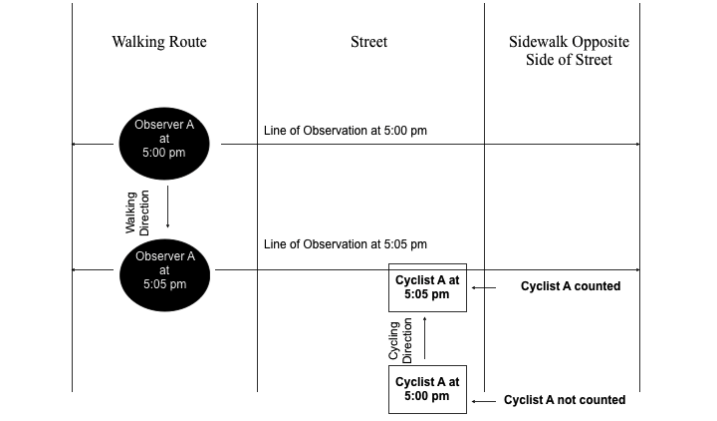
Block Walk Method procedure.

During an observation period, 2 trained observers (1 wearing a WVD and the other not wearing a WVD) will traverse an observation route at a pace of 100 ft/min (50 steps/min [largo]; stride width 2 ft; pace set by a battery-powered metronome). The observer without the WVD will record the number of individuals engaging in the targeted activities within an observation field. The observation field will be defined as a line extending to the left and right of the observer’s shoulders, linear and perpendicular from the observer’s plane of motion. The observation fields are expected to range in width from 30 to 70 ft. and include both sidewalks (if present) and the streets associated with an observation route. Individuals will be counted only if they cross a parallel plane of motion with the observer (Figure 1). For example, individuals walking down the sidewalk toward the observer (from ahead or from behind the observer) are counted if they continue to walk past the observer. An individual will be counted only once in an observation route on a given day of observation. When an observer encounters a street intersecting an observation route being observed, they will cease observing, cross the street, and then resume observations. An observation recording instrument was previously developed specifically for the procedure [32]. The instrument was designed so that an observer could record the PA observed, the street name where the PA occurred, and the number of individuals engaged in the PA. The instruments will contain information specific to each neighborhood including detailed walking directions and a map (*note: We decided not to use a single observer to conduct the BWM while wearing the WVD because the BWM requires an observer to look away from the observation field while entering data on the BWM instrument, and we have found this to be a source of error especially with larger groups. This is a deficit we expect the WVD to rectify*).

#### Outcome Variable

The primary outcome variable for aim 1 is the number of individuals observed walking, cycling, running, and standing/sitting along each observation route/50 min of observation.

#### Manual Video Analysis

The 2 study’s principal investigators will conduct independent evaluations of the videos obtained during the BWM. This will be done over a 1-year period beginning after the first week of BWMs are completed. They will use the BWM criteria to count individuals walking, cycling, running, and sitting/standing on sidewalks and streets along the observation routes.

#### Observer Training

All observers will participate in 2 training sessions before beginning data collection. During the first training session, they will be given detailed instructions on the BWM and procedures to be used. The second training session will involve mock field observations.

#### Meteorological Conditions

Data on meteorological conditions (rainfall, relative humidity, temperature, wind speed, and barometric pressure) for the exact time of day observations are conducted will be obtained from an automated weather sensor system located at the local airport.

#### Wearable Video Device—Pivothead Smart

**Figure 2:**
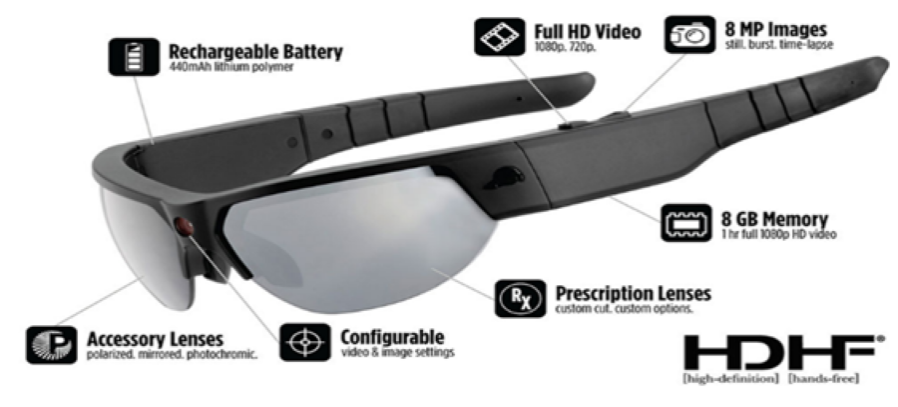
The Pivothead sunglasses used in this study.

**Figure 3:**
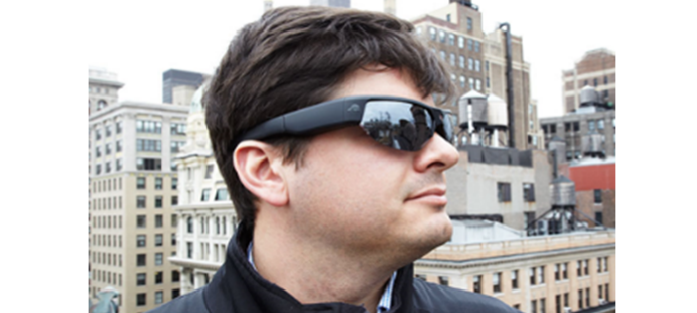
Example of Pivothead sunglasses being worn.

**Figure 4:**
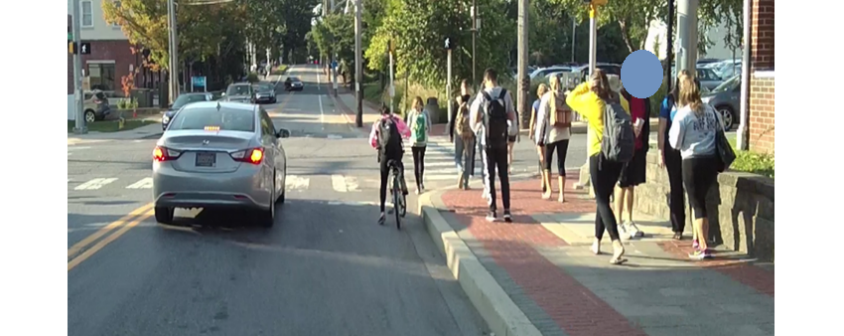
High resolution image taken with Pivothead glasses.

The Pivothead Smart (Pivothead, Denver, CO) is a state-of-the-art, noninvasive WVD indistinguishable from a pair of normal sunglasses (Figures 2 and 3). The camera is discretely centered in the bridge of the glasses for the truest first-person perspective possible, and it features an 8 MP Sony complementary metal-oxide-semiconductor sensor for capturing full 1080 p high definition 4 mega-pixel video at 30 frames per second as well as 8 mega-pixel stills (Figure 4). The glasses accept a 32 GB memory card allowing up to 8 hours of video recording per card at 1080 p. They have a self-contained battery providing 6 to 8 hours of recording time; a 77 degree field of vision, which approximates the human 90 degree field of vision; and they can be fitted with polarized prescription lenses. The Pivothead also allows for audio recordings (helpful for obtaining auxiliary information), time and date stamp, and geolocation capabilities, which can be used to create and retain precise maps of the observation routes.

### Aim 2

#### Observation Routes

We will use the videos of observation routes assessed in aim 1.

#### Procedures (Video Analysis)

The WVD video data, along with annotated ground truth for each human and feature of interest, will be analyzed automatically using multiple deep CNNs. The first deep CNN will be used in collaboration with the Simple, Online, and Real-time Tracking algorithms to determine the number of humans in BWM videos who cross the path of the observer (criteria for being counted) and the distance they traveled per unit time before crossing paths with the observer. For each human in the video, a bounding box will be drawn around their pixels, with identifying information such as faces blurred automatically. Once the humans in the scene are identified, activity recognition will be the next step. Activities will include standing/sitting, walking, cycling, and running. For bicycle riders, the answer is already given by the detection algorithm. For other activities, a new, separate deep network can be applied to classify the target behavior. An activity is a temporal event that is defined across many frames, so a recurrent neural network will need to be designed to handle this. These networks must be tested and fine-tuned for ground-level views. There are several state-of-the-art networks to choose from, but because of the dynamic nature and heterogeneous viewpoints, a new network architecture may be necessary. The output of the automatic methods can be compared against ground truth to give an accuracy score for how reliable the automatic methods are.

#### Outcome Variable

The primary outcome variable for aim 2 is the number of individuals observed walking, cycling, running, and sitting/standing along each observation route/50 mins of observation.

#### Statistical Analysis

Before developing statistical models, an examination of the univariate distribution of variables will be conducted (eg, scatter plots). Statistics such as means or proportions, SEs, ranges, and estimates of skewness and kurtosis will be derived. Data transformation procedures (eg, logarithmic) may be applied to quantitative variables whose distribution shows considerable departure from normality. Bland-Altman plots will be used to assess agreement on quantitative measures between the traditional BWM and WVD manual video analysis, the WVD manual video analysis, and the automated video analysis [53,54]. The difference between 2 methods for a variable of interest will be plotted against the average of the 2 methods for that variable. Horizontal lines representing the bias between 2 methods will be drawn at the mean difference. Additional horizontal lines will be drawn at the 95% limits of agreement (mean difference ± 1.96 [SD of the difference]). Two-way random effects intraclass correlation coefficients (ICCs) will also be calculated to examine agreement on quantitative measures between methods [55]. To test whether environmental variables (eg, obstructions such as trees) moderated outcome measure associations between methods, unadjusted and adjusted (for covariates) regression models that include a methods variable (eg, traditional BWM vs WVD-BWM), potential moderators, and interaction terms will be created to predict the outcome variable of interest (eg, number observed). In addition, we also will stratify the data by the levels of the moderator and re-examine effects. Simple effects analyses will be used to deconstruct significant interactions by examining associations between method and outcomes in separate subsamples stratified by levels of the moderator variables [56]. All statistical analyses will be performed using the SPSS statistical software package (IBM Corp Released 2015. IBM SPSS Statistics for Windows, version 23.0. IBM Corp).

## Results

Our research team has published 3 peer-reviewed journal articles examining the use of the BWM. In the first study, the BWM was used in 12 urban US census block groups to record the number of individuals walking, cycling, and running on sidewalks and streets and the geographical location (address) where they were observed [32]. The level of agreement between independent observers was >98% (530/538) for the PA type recorded. The number of individuals observed was correlated with US census block group characteristics (eg, percent walking/cycling to work) and weather (eg, temperature).

As the first study was limited to urban areas, we conducted a second study of the BWM in suburban settings [33]. Following the exact same procedures as in the first study, trained observers simultaneously walked along suburban sidewalks and streets while making independent recordings of the number of individuals walking/cycling/running and the address where the activity occurred. Analyses indicated that levels of agreement were 97.7% (347/355) for the address where an activity was observed, 94.6% (336/355) for PA type, and 89.3% (317/355) for the number performing an observed PA. Cohen kappa was .85 for address (*P*<.001), Cramer V was .89 for PA type (*P*<.001), and the ICC value was .85 (*F*_1,354_=6.64; *P*<.001) for the number performing an observed PA.

The third study was designed to determine if PAs observed using the BWM were associated with environmental characteristics [34]. A total of 14 environmental characteristics of 60, 1000 ft. long sidewalk and street observation routes, located in an urban, residential setting, were directly measured using standardized procedures, and the number of individuals walking, running, and cycling along the routes were assessed with the BWM. A total of 473 individuals were observed during 3600 total min of observation with 315 walking, 116 cycling, and 42 running. A greater number of individuals were seen walking along routes having more traffic, sidewalk defects, graffiti, and litter and poor property aesthetics. Only 1 environmental characteristic was associated with cycling, and none were significantly related with running.

We have previously deployed CNNs to detect cars, bikes, and pedestrians at busy intersections in collaboration with the Delaware Department of Transportation. Using a GoPro Hero Silver 3 with 720 p resolution at 30 fps, videos of pedestrians and cars were recorded over the course of a few hours. Using a modification of You Only Look Once with additional postprocessing, pedestrians, bicycle riders, and cars were automatically and accurately detected from the video (97% agreement with human detection). Tracking was performed with the Simple, Online, and Real-time Tracking algorithm, which uses a deep network for feature extraction and matching and a Kalman filter to improve the reliability [57,58].

## Discussion

### General

Efforts to increase PA are needed to reach a large portion of the population, and community-level interventions are highly recommended for this purpose. To accurately assess their effectiveness, the proposed study is being conducted to develop a new BWM that uses current technology to capture and analyze video data for the purpose of measuring PAs performed on sidewalks and streets. At this study’s completion, we will have demonstrated that a WVD can be used to improve the acquisition and accuracy of data collected using the BWM and that machine learning and recognition software can be used to automatically extract information on PAs occurring on the sidewalks and streets from the videos.

The outcomes from this study have the potential to establish new levels of accuracy for measuring PA on sidewalks and streets and advance the study of PA by using machine learning (deep CNNs) to automatically extract relevant data from the videos. In addition, the proposed study will lead to further developments in this area that will allow for other important characteristics captured by the WVD to be determined with deep CNNs including geographical-level (eg, street segment and park) caloric expenditure, demographics (eg, sex and age), health status (eg, body mass index) as well as current environmental conditions that could affect PA (eg, acts of incivility and weather). Therefore, the potential exists for this study to not only create a novel and valuable tool for researchers but develop an approach that could be easily used by public health officials, government agencies, and numerous other community groups.

### Potential Problems and Solutions

#### Mechanical Failure

We expect the WVD to experience technical difficulties at times. In recent months, we have been working with the Pivothead Smart, and on a few occasions there were issues with the recording device stopping during use and uploading videos from the device to a computer, which was because of a faulty cable. To correct or minimize these issues, we will provide observers with a reserve pair of glasses and keep additional cables on hand.

#### Safety Concerns

It is probable that some observations will be conducted in high-crime areas, making it unsafe for data collectors. We have encountered this in previous studies and addressed this by having a law enforcement officer accompany data collectors when necessary.

#### Hawthorne Effect

The Hawthorne effect is the alteration of behavior by the subjects of a study because of their awareness of being observed. Although this is a valid concern, in our past studies using the BWM we have not found any noticeable reaction to the observers. This is likely because of a couple of reasons such as the observers not standing out and appearing simply as individuals walking down the sidewalk. If people do react to the observers, it would most likely be because the observers walk at a slow pace and periodically write in a notebook while walking. We expect this concern to be eliminated with the use of the video glasses that are indistinguishable from regular glasses.

## Appendix

Multimedia Appendix 1 City characteristics.

Multimedia Appendix 2 NIH Summary Statement.

## Abbreviations

BWM: Block Walk Method
CNN: convolutional neural network
GPS: global positioning system
ICC: intraclass correlation coefficient
PA: physical activity
WVD: wearable video device
