# Supplementary figures and images for: Assessing Physical Activities Occurring on Sidewalks and Streets: Protocol for a Cross-Sectional Study
Source: JMIR Res Protoc. 2019 Jul 30;8(7):e12976. doi: 10.2196/12976 (PMC6692107; doi:10.2196/12976)

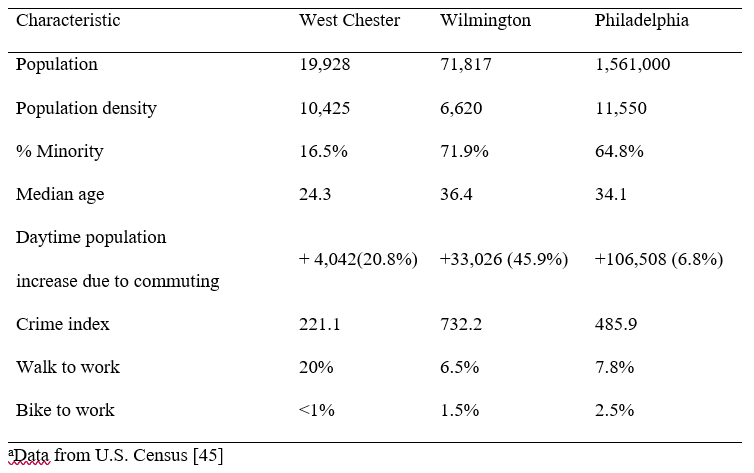

Supplement: Multimedia Appendix 1 [file resprot_v8i7e12976_app1.png]
