# Supplementary material for: Assessing Physical Activities Occurring on Sidewalks and Streets: Protocol for a Cross-Sectional Study
Source: JMIR Res Protoc. 2019 Jul 30;8(7):e12976. doi: 10.2196/12976 (PMC6692107; doi:10.2196/12976)
Supplement: Multimedia Appendix 2 [file resprot_v8i7e12976_app2.docx]

**PROGRAM CONTACT:**

**Josephine Boyington 301-594-2542**

[**boyingtonje@mail.nih.gov**](mailto:boyingtonje@mail.nih.gov)

**SUMMARY STATEMENT**

**( Privileged Communication )**

***Release Date:* 02/22/2018**

***Revised Date:***

**Principal Investigators (Listed Alphabetically): DOMINICK, GREGORY MITCHELL**

**SUMINSKI, RICHARD ROBERT (Contact)**

***Application Number:* 1 R21 HL143553-01**

**Applicant Organization: UNIVERSITY OF DELAWARE**

***Review Group:* KNOD**

**Kidney, Nutrition, Obesity and Diabetes Study Section**

***Meeting Date:* 02/01/2018 *RFA/PA:* PAR15-171**

***Council:* MAY 2018 *PCC:* HHCP N**

***Requested Start:* 07/01/2018 *Dual PCC:* RAJ DUAL**

***Dual IC(s):* DK**

***Project Title:* A cutting edge approach to assessing physical activities occurring on sidewalks/streets**

***SRG Action:* Impact Score:22 Percentile:3 +**

***Next Steps:* Visit https://grants.nih.gov/grants/next_steps.htm Human Subjects: 48-At time of award, restrictions will apply**

**Animal Subjects: 10-No live vertebrate animals involved for competing appl.**

**Gender: Minority: Children:**

**1A-Both genders, scientifically acceptable**

**1A-Minorities and non-minorities, scientifically acceptable 1A-Both Children and Adults, scientifically acceptable Clinical Research - not NIH-defined Phase III Trial**

| **Project Year** |  | **Direct Costs Requested** |  | **Estimated Total Cost** |
| --- | --- | --- | --- | --- |
| **1** |  | **150,000** |  | **232,022** |
| **2** |  | **125,000** |  | **193,352** |
| **TOTAL** |  | **275,000** |  | **425,374** |

**ADMINISTRATIVE BUDGET NOTE: The budget shown is the requested budget and has not been adjusted to reflect any recommendations made by reviewers. If an award is planned, the costs will be calculated by Institute grants management staff based on the recommendations outlined below in the COMMITTEE BUDGET RECOMMENDATIONS section.**

## 1R21HL143553-01 SUMINSKI, RICHARD

**PROTECTION OF HUMAN SUBJECTS UNACCEPTABLE**

**RESUME AND SUMMARY OF DISCUSSION:** The proposed study will develop and test a wearable video device and computer video analysis system to assess physical activity performed on sidewalks and streets. The premise that existing methodology for counting pedestrians (BWM) is limited and new video analysis and machine learning techniques is appropriate is well established by the preliminary studies demonstrating the accuracy of the BWM (high level of inter-rater agreement), and use of machine learning techniques to identify pedestrians, bicycle riders, and cars with 97% agreement to human detection. However, the premise that counting physical activity on streets and sidewalks will provide critical information to meaningfully impact individual level physical activity (without otherwise influencing individual behavior) is not well supported in this application. The current manual approach is time consuming and prone to errors when large groups of individuals are encountered, or encounters occur at a high frequency, therefore this system could be a significant improvement in more accurate and low burden evaluation of the impact of environmental conditions (e.g. traffic) on walking and biking activities. A minor to moderate concern was raised about the lack of attention to how data collectors might influence behavior, especially in those instances when data collectors are accompanied by officers. Others also thought that the dependency on hardware is not amenable to scalability, though the majority felt that this was minor as this is a necessary exploratory first step. The approach to machine learning is innovative and rigor and reproducibly are well addressed with a sound analytic plan ensuring robust findings. Others did not feel that the issue of walkability especially in neighborhoods of those most vulnerable was adequately addressed and they thought that this renders the findings less generalizable. The investigative team is strong, with experience in the proposed areas of research, though some on the panel have concerns about overlap and whether there is sufficient expertise to perform the machine learning approaches proposed. At the end of the discussion, the panel concluded that the potential impact on the field of physical activity and neighborhood environment is likely to be high.

**DESCRIPTION (provided by applicant):** A considerable proportion of outdoor physical activity is done on sidewalk/streets. For example, we found that ~70% of adults who walked during the previous week used the sidewalks/streets around their homes. Interventions conducted at geographical levels (e.g., community) and studies examining relationships between environmental conditions (e.g., traffic) and walking/biking, necessitate a reliable measure of physical activities performed on sidewalks/streets.

The Block Walk Method (BWM) is one of the more common approaches available for this purpose. Although it utilizes reliable observation techniques and displays criterion validity, it remains relatively unchanged since its introduction in 2006. It is a non-technical, labor-intensive, first generation method. Advancing the BWM would contribute significantly to our understanding of physical activity behavior. Therefore, the objective of the proposed study is to develop and test a new BWM that utilizes a wearable video device (WVD) and computer video analysis to assess physical activities performed on sidewalks/streets. The following aims will be completed to accomplish this objective. Aim 1: Improve the BWM by incorporating a WVD into the methodology. The WVD is a pair of eyeglasses with a high definition video camera embedded into the frames. We expect the WVD to be a viable option for improving the acquisition and accuracy of data collected using the BWM. Aim 2: Advance the WVD- enhanced BWM by applying machine learning and recognition software to automatically extract information on physical activities occurring on the sidewalks/streets from the videos. Methods: Trained observers (one wearing and one not wearing the WVD) will walk together at a set pace along predetermined, 1000 ft. sidewalk/street segments representing low, medium, and high walkable areas.

During the walks, the non-WVD observer will use the traditional BWM to record the number of individuals standing/sitting, walking, biking, and running along the segments. The WVD observer will only record a video while walking. Later, two investigators will view the videos to determine the numbers of individuals performing physical activities along the segments. For aim 2, the video data will be analyzed automatically using multiple deep convolutional neural networks (CNNs) to determine the number of humans in a segment as well as the type of physical activities being performed. Bland Altman methods and intraclass correlation coefficients will be used to assess agreement. Potential sources of error such as occlusions (e.g., trees) will be assessed using moderator analyses. We expect the new approach will enhance measurement accuracy while reducing the burden of data collection. In the future, we will expand the capabilities of the WVD-CNNs system to allow for the determination of other characteristics captured by the videos such as caloric expenditure and environmental conditions. Our long-term goal is to substantially improve the assessment of physical activity and our understanding of physical activity behavior.

**PUBLIC HEALTH RELEVANCE**: Sidewalks and streets are public spaces where a considerable amount of physical activity occurs. As such, accurate and feasible methods are important for understanding the role these areas play in facilitating physical activity and improving public health. In the proposed study, we will develop and test a state-of-the-art, observation approach using a wearable video device and computer technology to measure physical activities performed on sidewalks/streets. Findings have the potential to significantly impact research in this area and inform the further development of technology-enhanced observation methods for widespread, practical applications that promote healthy lifestyles and reduce chronic disease risks across the age spectrum.

## CRITIQUE 1

Significance: 2

Investigator(s): 3

Innovation: 1

Approach: 3

Environment: 1

**Overall Impact:** The goal of this application is to develop and test a wearable video device and computer video analysis system to assess physical activity performed on sidewalks and streets. A high-resolution video device would be embedded in a pair of glasses worn by the researcher. The investigators propose to advance the analysis system using machine learning to automatically identify

different physical activities occurring on the sidewalks or streets. Currently this is done manually, which is time consuming and prone to errors when large groups of individuals are encountered, or encounters occur at a high frequency. This system would be useful in studies performed in the community and in studies assessing the impact of environmental conditions (e.g. traffic) on walking and biking activities.

If successful, this may produce a system that reduces the burden of data collection in this type of study. The premise of the research is based on the observation that access to sidewalks and streets is associated with greater physical activity levels, and that this is impacted by the built environment. The premise is supported by preliminary studies demonstrating the accuracy of the WBM (high level of inter- rater agreement), and use of machine learning techniques to identify pedestrians, bicycle riders, and cars with 97% agreement to human detection. Using machine learning to identify activities from video is a highly innovative aspect of this proposal. Both men and women will be measured, as will children, but sex is not considered as a biological variable; this is appropriate for this type of research. Rigor and reproducibility is addressed by repeated measurements with trained observers. The major score driving factors the high level of innovation, robust approach, and high level of significance. The investigative team is strong, although there are some minor concerns about overlap and whether there is sufficient expertise to perform the machine learning approaches proposed. This type of system could

have a substantial impact on the field of physical activity assessment as the methods could be applied to any study requiring direct observation methods. Overall, my enthusiasm for this application is high.

## Significance: Strengths

- - Assessing free-living physical activity using direct observation methods is time consuming and requires highly trained observers. Advancing a system that could record and analyze PA automatically in these settings would be a great advancement in measurement techniques. The reach of the impact potentially extends to any study requiring direct observation.
  - The premise of the research is based on the observation that access to sidewalks and streets is associated with greater physical activity levels, and that this is impacted by the built environment. The premise is supported by preliminary studies demonstrating the accuracy of the WBM (high level of inter-rater agreement), and use of machine learning techniques to identify pedestrians, bicycle riders, and cars with 97% agreement to human detection.

## Weaknesses

- - None noted

## Investigator(s): Strengths

- - The PI (Suminski) has created the original block walk method, and has performed several studies utilizing this approach to assess physical activity that occurs in outdoor settings, as well as the impact of the built environment. He is trained in exercise physiology and epidemiology, and several publications relating to measuring physical activity and studying the impact of the built environment
  - Dr. Saponaro is a post-doc with training in computer science. He will oversee the machine learning aspects of this proposal
  - Dr. Babodt will provide statistical support

## Weaknesses

- - Dr. Dominick’s expertise overlaps with the PI; his individual contributions to the project are not well defined
  - It is not clear if Dr. Sapanaro has related experience in performing the machine learning approaches to be applied in this study.

## Innovation: Strengths

- - Using machine learning to identify activities from video is highly innovative. This may have impact in studies relying on direct observation.
  - Has the potential to capture elements of the built environment that may moderate the effects of sidewalk access on PA (not an aim of this study).

## Weaknesses

- - None noted

## Approach:

**Strengths**

- - Comparison of the wearable video system to manual observation with the BWM in small, medium and large cites, and within 3 different neighborhoods in each city with various levels of walkability. Different segments in each city will be measured
  - Reproducibility will be addressed by multiple measurements of each segment.
  - Observations will be performed on weekdays and weekend days, and at various times

## Weaknesses

- - The wearable glasses have a 77º field of view vs. 90º for normal human vision. Will this be accounted for in terms of determining accuracy? This is a minor weakness.

## Environment:

**Strengths**

- - The facilities and resources at the University of Delaware, including the video imaging lab, provide the support necessary to complete the proposed studies

## Weaknesses

- - None noted

## Protections for Human Subjects:

Acceptable Risks and/or Adequate Protections

Data and Safety Monitoring Plan (Applicable for Clinical Trials Only): Acceptable

## Inclusion of Women, Minorities and Children:

- - Sex/Gender: Distribution justified scientifically
  - Race/Ethnicity: Distribution justified scientifically
  - For NIH-Defined Phase III trials, Plans for valid design and analysis:
  - Inclusion/Exclusion of Children under 18: Including ages <18; justified scientifically

## Vertebrate Animals:

Not Applicable (No Vertebrate Animals)

## Biohazards:

Not Applicable (No Biohazards)

## Resource Sharing Plans:

Acceptable

## Authentication of Key Biological and/or Chemical Resources:

Not Applicable (No Relevant Resources)

## Budget and Period of Support:

Recommend as Requested

## CRITIQUE 2

Significance: 2

Investigator(s): 3

Innovation: 1

Approach: 2

Environment: 1

**Overall Impact:** The proposed project will improve an existing methodology to collect street-based pedestrian activity (Block Walk Method, BWM) using modern tools, such as wearable video devices (WVD) for data collection and machine learning for automated data analysis. Understanding the numbers of pedestrians using streets has the potential to provide empirical data for city planners and for interventions to increase street based physical activity. However, such interventions have the potential to influence some individuals who may be inclined to exercise outside on streets. While the tool itself might be useful for counting pedestrians, other efforts are likely needed to reach a large portion of the population to increase overall physical activity. The premise that the existing methodology for counting pedestrians (the BWM) has limitations that can potentially be overcome using new video analysis and machine learning techniques is well established in the presented preliminary studies and literature. The investigative team has experience in the proposed areas of research. The proposed project scored high in innovation for the data collection and analysis tools. Weaknesses in approach include lack of representation of population in selection of measurement areas, lack of detail on how data will be handled in the case of mechanical failure, while strengths include selection of streets for data collection based on walkability and replication of manual evaluators for data analysis. Score driving factors were some limitations in the approach.

## Significance:

**Strengths**

- - The significance of the proposed project lies in the development of new tools to efficiently count pedestrians on sidewalks and streets, which has implications for city planning and potentially for street-based physical activity.
  - The premise that the existing methodology for counting pedestrians (the BWM) has limitations that can potentially be overcome using new video analysis and machine learning techniques is well established in the presented preliminary studies and literature.

## Weaknesses

- - Ultimately what is learned in the proposed project has potential to influence a portion of the population who may be inclined to commute or exercise outside on streets. While the tool itself might be useful for counting pedestrians, other efforts are likely needed to reach a large portion of the population to increase overall physical activity.

## Investigator(s):

**Strengths**

- - Suminski has experience in physical activity measurement and Dominick has a masters in exercise physiology. The team has experience working with the proposed measurement

techniques, including computer science/video analysis and has published on the use of the BWM. Thus, the team is well equipped to address the proposed research questions.

## Weaknesses

- - The MPI plan does not lay out a plan for conflict resolution using neutral parties. The main protocol relies on the project personnel rather than neutral parties

## Innovation:

**Strengths**

- - The investigators propose novel techniques to measure sidewalk/street-based pedestrian counts using WVD. The proposed project has the potential to shift current data collection practices.
  - The investigators also propose novel analytical techniques to process and extract the video- based data in automated fashion.

## Weaknesses

- - None noted

## Approach:

**Strengths**

- - The selection of cities and stratification by walkability using Walk Score are well thought out to provide variability for walkability of streets relative to measurement of pedestrian counts.
  - Use of independent evaluators for manual video analysis is a robust approach.
  - The pivot head provides high resolution video.

## Weaknesses

- - While the sampled areas will represent different types of walkability and the selected cities are varied in SES and minority population, it does not seem that the investigators will sample areas for data collection based on population characteristics, such as SES or minority status, thus it is unclear whether the resulting information will be population-representative.
  - It is unclear whether the two observers walking the streets (and in the case of the accompaniment with law enforcement officers) will itself influence street based pedestrian activity.
  - While the potential for mechanical failure is noted and extra devices and cables will be used, it is unclear how the data collection relative to mechanical failure will be handled. How will decisions be made on whether or not to include data surrounding identification of mechanical failure.
  - Although not directly proposed, the future directions in terms of measuring caloric expenditure using this technology is likely beyond the scope of the proposed tools. While this does not impact the score for the proposed project, the investigators are cautioned not to over interpret the resulting video data for caloric expenditure without considerable validation.

## Environment:

**Strengths**

- - The University of Delaware has excellent resources to support the proposed project, the robotics and control laboratory provides specific resources to support the proposed project.

## Weaknesses

- - None noted

## Protections for Human Subjects:

Acceptable Risks and/or Adequate Protections

- - While the application indicates that faces will be blurred, it is likely that the original videos of recognizable individuals will exist somewhere and there should be some mechanism to protect individuals who are captured in the file that comes directly from the field before blurring.

Data and Safety Monitoring Plan (Applicable for Clinical Trials Only): Not Applicable (No Clinical Trials)

## Inclusion of Women, Minorities and Children:

- - Sex/Gender: Distribution justified scientifically
  - Race/Ethnicity: Distribution justified scientifically
  - For NIH-Defined Phase III trials, Plans for valid design and analysis: Not applicable
  - Inclusion/Exclusion of Children under 18: Including ages <18; justified scientifically
  - As the proposed project entails video recordings of streets, all individuals present on streets regardless of age, sex, race will be included. However, street segments are selected for walkability not population representation.

## Vertebrate Animals:

Not Applicable (No Vertebrate Animals)

## Biohazards:

Not Applicable (No Biohazards)

## Resource Sharing Plans:

Acceptable

## Authentication of Key Biological and/or Chemical Resources:

Not Applicable (No Relevant Resources)

## Budget and Period of Support:

Recommended budget modifications or possible overlap identified:

- - It seems as though only personnel effort for researchers is included in the budget. It is unclear whether the team has access to the pivothead devices. The number, rate, and amount of effort for data collectors is not specified. Thus, the feasibility of the proposed project is unclear.

## CRITIQUE 3

Significance: 3

Investigator(s): 2

Innovation: 3

Approach: 4

Environment: 3

**Overall Impact:** The application proposes to develop and test the augmentation of the Block Walk Method with a wearable video device coupled with video analysis to assess physical activity on sidewalks. The innovation is in the use of an eye glasses equipped with a video camera to continuously capture PA on sidewalks. In addition, the proposal utilizes machine learning to extract information on PA occurring in sidewalks. The study will explore geographical level energy expenditure and the utility of the technology in other outdoor areas like parks. A cross sectional study will be conducted stratifying samples to cover a wide range of conditions related to city size and walkability. The premise of this research that video technology that is unobtrusive can significantly improve the BWM method which is supported by preliminary work as well. The significance of this work is high as it will enable more accurate data collection and lower burden in data collection.

## Significance:

**Strengths**

- - Extending BWM with video technology provides an opportunity to replace tedious and error prone observational methods. Data can also be further processed using advanced machine learning techniques to provide deeper analyses and results.
  - There is existing evidence that video annotation provides a more detailed opportunity to analyze a scene and therefore, the premise of this work is plausible and reasonable.

## Weaknesses

- - There are many cities in the country where walking on sidewalks is uncommon. This project will not influence these areas.

## Investigator(s):

**Strengths**

- - Excellent team, the PI Dr. Suminski was key in the development of the BWM. Dr. Dominik has ample experience managing PA measurement studies and Dr. Saponaro is a machine learning expert who will develop the video processing software. It is very clear in the preliminary work section that the team can execute this study successfully**.**

## Weaknesses

- - None noted.

## Innovation:

**Strengths**

- - The project is the first to use camera equipped glasses to enhance the Block Walk Method (BWM). The use of the glasses mounted camera is innovative and can allow for other factors to be measured such as energy expenditure, demographics, health status (BMI),

## Weaknesses

- - None noted.

## Approach:

**Strengths**

- - Samples are stratified to allow for different walking conditions
  - Walkscore will be used to evaluate the walkability of different neighborhoods.
  - Video will be analyzed manually and multiple raters will be used to provide accurate annotations.
  - Pivothead Smart is an advanced video device that will be used to provide high resolution imagery which will enable a variety of analyses including activity type classification.

## Weaknesses

- - It is not clear how the deployment and data capture process will work
  - It is not clear how the authors intend to estimate metrics such as caloric expenditure from videos

## Environment:

**Strengths**

- - University of Delaware is well positioned to conduct this research with a strong engineering department and multiple engineering labs for prototyping.

## Weaknesses

- - None noted.

## Protections for Human Subjects:

Data and Safety Monitoring Plan (Applicable for Clinical Trials Only):

## Inclusion of Women, Minorities and Children:

- - Sex/Gender:
  - Race/Ethnicity:
  - For NIH-Defined Phase III trials, Plans for valid design and analysis:
  - Inclusion/Exclusion of Children under 18:

## Vertebrate Animals:

Not Applicable (No Vertebrate Animals)

## Biohazards:

Not Applicable (No Biohazards)

## Resource Sharing Plans:

Acceptable

## Authentication of Key Biological and/or Chemical Resources:

Not Applicable (No Relevant Resources)

## Budget and Period of Support:

Recommend as Requested

## CRITIQUE 4

Significance: 2

Investigator(s): 1

Innovation: 1

Approach: 2

Environment: 2

**Overall Impact:** This application proposes to improve upon an existing paper street-level audit of public physical activity engagement using wearable video technology and subsequent automated visual analysis methods. Physical activity is important to health across the lifespan. The study builds off the premise that strategies to increase widespread commuting-related and recreational physical activity may have important population-level impact. Street-level audits are a source of information contributing to the study of environmental supports of physical activity. This work builds directly off the principal investigator’s previous success in developing and evaluating the specified paper tool. If the aims of this study are achieved, this research will improve the original paper tool by providing a feasible, convenient, and valid approach to video auditing streets/sidewalks. An unanswered question is the general sustainability of this approach given planned obsoletion of technology inherent to manufacturers’ business plans. A device agnostic approach to video recording the surveilled area would be valuable. The approach as outlined is well thought out and obviously benefits from a deep understanding of issues that comes from a robust familiarity with described procedures. The investigative team is appropriate to meet the study aims as is the environment/facilities. The strengths of this proposal outweigh the minor weaknesses identified and therefore its overall impact is considered to be high.

## Significance:

**Strengths**

- - Scientific premise is that built environmental approaches to increasing physical activity in the community are most viable in terms of reach and impact.
  - Sidewalks/streets are common location of physical activity.
  - Audits are useful for surveillance, inference testing, and evaluation of program/policy

## Weaknesses

- - None noted.

## Investigator(s):

**Strengths**

- - Appropriate experience and training, good range of expertise on identified key personnel
  - Research builds on previous experience developing and evaluating pen-and-paper instrument

## Weaknesses

- - None noted.

## Innovation:

**Strengths**

- - Obvious innovation in wearable video recording technology and novel analytical approaches

## Weaknesses

- - A pen-and-paper audit already exists. This technological improvement seems more related to convenience, feasibility, potential reach, than an improvement in validity
  - Manufacturers’ business plans (e.g., planned obsoletion) put sustainability of developed technology in question

## Approach:

**Strengths**

- - Previous experience with proposed methods is apparent. This has enabled researchers to present their approach clearly, building off previous knowledge.
  - For example, they have the foresight to build in redundancy of measurements to improve reliability, including planning “back up” equipment in case of failure
  - There is a well thought out strategy to reduce ordering, seasonal effects; analysis is well planned and described. Again, apparent this builds off of previous successful work developing and evaluating the pen-and-paper tool

## Weaknesses

- - They indicate that they will engage local law enforcement agencies as needed for safety, however, there is no evidence (e.g., letter of support) to indicate that this is feasible (who pays costs for this service?)
  - They indicate that descriptive variables will not be captured, however, race/gender/weight can be estimated from investigator review of videos. This potential is not described.

## Environment:

**Strengths**

- - Appears to be adequate for the study needs

## Weaknesses

- - There is repetitive information provided in Facilities/Other Resources/Equipment section

## Protections for Human Subjects:

Acceptable Risks and/or Adequate Protections

- - Acceptable

Data and Safety Monitoring Plan (Applicable for Clinical Trials Only): Not Applicable (No Clinical Trials)

## Inclusion of Women, Minorities and Children:

- - Sex/Gender: Distribution justified scientifically
  - Race/Ethnicity: Distribution not justified scientifically
  - For NIH-Defined Phase III trials, Plans for valid design and analysis: Not applicable
  - Inclusion/Exclusion of Children under 18: Including ages <18; justified scientifically
  - Race/gender/weight can be estimated from investigator review of videos but this is not described

## Vertebrate Animals:

Not Applicable (No Vertebrate Animals)

## Biohazards:

Not Applicable (No Biohazards)

## Resource Sharing Plans:

Acceptable

## Authentication of Key Biological and/or Chemical Resources:

Not Applicable (No Relevant Resources)

## Budget and Period of Support:

Recommend as Requested

## THE FOLLOWING SECTIONS WERE PREPARED BY THE SCIENTIFIC REVIEW OFFICER TO SUMMARIZE THE OUTCOME OF DISCUSSIONS OF THE REVIEW COMMITTEE, OR REVIEWERS’ WRITTEN CRITIQUES, ON THE FOLLOWING ISSUES:

**PROTECTION OF HUMAN SUBJECTS: UNACCEPTABLE**

Although the application indicates that faces will be blurred, it is likely that the original videos of recognizable individuals will exist somewhere, but the application does not articulate a mechanism to protect individuals who are captured in the files before blurring occurs.

## INCLUSION OF WOMEN PLAN: ACCEPTABLE INCLUSION OF MINORITIES PLAN: ACCEPTABLE INCLUSION OF CHILDREN PLAN: ACCEPTABLE

**COMMITTEE BUDGET RECOMMENDATIONS: The budget was recommended as requested.**

Footnotes for 1 R21 HL143553-01; PI Name: SUMINSKI, RICHARD Robert

1 R21 HL143553-01 14 KNOD SUMINSKI, R

# + Derived from the range of percentile values calculated for the study section that reviewed this application.

NIH has modified its policy regarding the receipt of resubmissions (amended applications). See Guide Notice NOT-OD-14-074 at <http://grants.nih.gov/grants/guide/notice-files/NOT-OD-> 14-074.html. The impact/priority score is calculated after discussion of an application by averaging the overall scores (1-9) given by all voting reviewers on the committee and multiplying by 10. The criterion scores are submitted prior to the meeting by the individual reviewers assigned to an application, and are not discussed specifically at the review meeting or calculated into the overall impact score. Some applications also receive a percentile ranking. For details on the review process, see [http://grants.nih.gov/grants/peer_review_process.htm#scoring.](http://grants.nih.gov/grants/peer_review_process.htm#scoring)

MEETING ROSTER

Kidney, Nutrition, Obesity and Diabetes Study Section Population Sciences and Epidemiology Integrated Review Group CENTER FOR SCIENTIFIC REVIEW

KNOD 02/01/2018 - 02/02/2018

Notice of NIH Policy to All Applicants: Meeting rosters are provided for information purposes only. Applicant investigators and institutional officials must not communicate directly with study section members about an application before or after the review. Failure to observe this policy will create a serious breach of integrity in the peer review process, and may lead to actions outlined in NOT-OD-14-073 at https://grants.nih.gov/grants/guide/notice-files/NOT-OD-14-073.html and NOT-OD-15-106 at

https://grants.nih.gov/grants/guide/notice-files/NOT-OD-15-106.html, including removal of the application from immediate review.

CHAIRPERSON(S) BRANDT, MARY L, MD *

GORDON-LARSEN, PENNY, PHD PROFESSOR

DEPARTMENT OF NUTRITION

GILLINGS SCHOOL OF GLOBAL PUBLIC HEALTH UNIVERSITY OF NORTH CAROLINA AT CHAPEL HILL CHAPEL HILL, NC 27599

PROFESSOR OF SURGERY, PEDIATRICS AND ETHICS AND INTERIM SENIOR ASSOCIATE DEAN OF MEDICAL EDUCATION

DEPARTMENT OF SURGERY TEXAS CHILDREN'S HOSPITAL BAYLOR COLLEGE OF MEDICINE HOUSTON, TX 77030

MEMBERS CARNETHON, MERCEDES RENEE, PHD

ALBINALI, FAHD PHD, PHD * CHIEF TECHNOLOGY OFFICER EVERYFIT INC.

DEPARTMENT OF ARCHITECTURE CAMBRIDGE, MA 02140

BAIER, LESLIE J, PHD CHIEF, GENOMICS UNIT

PHOENIX EPIDEMIOLOGY CLINICAL RESEARCH BRANCH NATIONAL INSTITUTE OF DIABETES AND DIGESTIVE AND KIDNEY DISEASES

NATIONAL INSTITUTES OF HEALTH PHOENIX, AZ 85004

BALLMAN, KARLA V, PHD * PROFESSOR AND CHIEF

DEPARTMENT OF HEALTH CARE POLICY AND RESEARCH

DIVISION OF BIOSTATISTICS AND EPIDEMIOLOGY

ASSOCIATE PROFESSOR

DEPARTMENT OF PREVENTIVE MEDICINE FEINBERG SCHOOL OF MEDICINE NORTHWESTERN UNIVERSITY

CHICAGO, IL 60611

CASSIDY-BUSHROW, ANDREA E, MPH, PHD ASSOCIATE SCIENTIST

RESEARCH EPIDEMIOLOGIST

DEPARTMENT OF PUBLIC HEALTH SCIENCES HENRY FORD HEALTH SYSTEM

DETROIT, MI 48202

CATELLIER, DIANE J, DRPH * SENIOR STATISTICIAN DEPARTMENT OF BIOSTATISTICS RTI INTERNATIONAL

WEILL CORNELL MEDICINE RESEARCH TRIANGLE PARK, NC 27709 NEW YORK CITY, NY 10065

BARNARD, JOHN, PHD * SECTION HEAD

DEPARTMENT OF QUANTITATIVE HEALTH SCIENCES CLEVELAND CLINIC

CLEVELAND, OH 44195

CHEN, KONG Y, PHD *

DIRECTOR, METABOLIC RESEARCH CORE DIVISION OF INTRAMURAL RESEARCH/ NIDDK NATIONAL INSTITUTES OF HEALTH

DIRECTOR, METABOLIC RESEARCH CORE, ENDOCRINE UNI BETHESDA, MD 20892

CORLEY, DOUGLAS ALLEN, MD, PHD * INVESTIGATOR

DIVISION OF RESEARCH KAISER PERMANENTE OAKLAND, CA 94612

COX, LAURA A, PHD * KAPLAN, ROBERT C, PHD

PROFESSOR OF INTERNAL MEDICINE ASSOCIATE DIRECTOR OF THE CENTER FOR PRECISION MEDICINE

MEDICAL CENTER BOULEVARD

WAKE FOREST UNIVERSITY HEALTH SCIENCES WAKE FOREST UNIVERSITY HEALTH SCIENCES WINSTON-SALEM, NC 27157

DE BOER, IAN H, MD ASSOCIATE PROFESSOR DIVISION OF NEPHROLOGY

AND KIDNEY RESEARCH INSTITUTE UNIVERSITY OF WASHINGTON SEATTLE, WA 98195

DUGAS, LARA RUTH, PHD * ASSISTANT PROFESSOR DIVISION OF EPIDEMIOLOGY

DEPARTMENT OF PUBLIC HEALTH SCIENCES LOYOLA UNIVERSITY CHICAGO

MAYWOOD, IL 60153

DUNAIF, ANDREA E, MD *

PROFESSOR AND VICE CHAIR FOR RESEARCH DEPARTMENT OF MEDICINE

NORTHWESTERN UNIVERSITY

PROFESSOR

DEPARTMENT OF EPIDEMIOLOGY AND POPULATION HEALTH

ALBERT EINSTEIN COLLEGE OF MEDICINE YESHIVA UNIVERSITY

BRONX , NY 10461

KOVESDY, CSABA PAL, MD * PROFESSOR

COLEMAN COLLEGE OF MEDICINE

UNIVERSITY OF TENNESSEE HEALTH SCIENCE CENTER MEMPHIS, TN 38163

LEMAS, DOMINICK JOSEPH, PHD * RESEARCH ASSISTANT PROFESSOR INSTITUTE OF CHILD HEALTH POLICY UNIVERSITY OF FLORIDA GAINESVILLE, FL 32608

MELANSON, EDWARD L, PHD * ASSOCIATE PROFESSOR

DIVISION OF ENDOCRINOLOGY, METABOLISM, AND DIVISION OF GERIATRIC MEDICINE

UNIVERSITY OF COLORADO, DENVER AURORA, CO 80045

CHICAGO, IL 60611 NATARAJAN, LOKI, PHD

PROFESSOR

GORAN, MICHAEL ISAAC, PHD PROFESSOR AND DIRECTOR DEPARTMENT OF PEDIATRICS UNIVERSITY OF SOUTHERN CALIFORNIA LOS ANGELES, CA 90033

GORDON, DEREK, PHD *

DEPARTMENT OF FAMILY MEDICINE AND PUBLIC HEALTH MOORES UNIVERSITY OF CALIFORNIA

SAN DIEGO CANCER CENTER LA JOLLA, CA 92093-0901

NAVAS-ACIEN, ANA, MD, PHD * PROFESSOR

ASSOCIATE PROFESSOR ENVIRONMENTAL SCIENCES

DEPARTMENT OF GENETICS SCHOOL OF ARTS AND SCIENCES RUTGERS UNIVERSITY PISCATAWAY TOWNSHIP, NJ 08854

HU, FRANK B, MD, MPH, PHD PROFESSOR

DEPARTMENT OF NUTRITION AND EPIDEMIOLOGY SCHOOL OF PUBLIC HEALTH

HARVARD UNIVERSITY

MAILMAN SCHOOL OF PUBLIC HEALTH COLUMBIA UNIVERSITY

NEW YORK, NY 10032

PETERSON, KAREN EILEEN, DSC PROFESSOR AND DIRECTOR

DEPARTMENT OF ENVIRONMENTAL HEALTH SCIENCES UNIVERSITY OF MICHIGAN

ANN ARBOR, MI 48109

BOSTON, MA 02115 PHELAN, SUZANNE, PHD *

PROFESSOR

HUNG, ADRIANA MPH, MD * DEPARTMENT OF KINESIOLOGY

STAFF PHYSICIAN AND CLINICAL INVESTIGATOR THE VA TENNESSEE VALLEY HEALTHCARE SYSTEM ASSOCIATE PROFESSOR OF MEDICINE VANDERBILT UNIVERSITY SCHOOL OF MEDICINE NASHVILLE, TN 37232

JOHN, DINESH, PHD * ASSISTANT PROFESSOR

DEPARTMENT OF HEALTH SCIENCES NORTHEASTERN UNIVERSITY BOSTON, MA 02115

CALIFORNIA POLYTECHNIC STATE UNIVERSITY SAN LUIS OBISPO, CA 93407

RANKINEN, TUOMO, PHD ASSOCIATE PROFESSOR

HUMAN GENOMICS LABORATORY PENNINGTON BIOMEDICAL RESEARCH CENTER BATON ROUGE, LA 70808-4124

SELVIN, ELIZABETH , PHD, MPH PROFESSOR

DEPARTMENTS OF EPIDEMIOLOGY AND MEDICINE JOHNS HOPKINS UNIVERSITY

BALTIMORE, MD 21287

COLEMAN, KAREN JACQUELINE, PHD RESEARCH SCIENTIST II

DEPARTMENT OF RESEARCH AND EVALUATION KAISER PERMANENTE SOUTHERN CALIFORNIA PASADENA, CA 91101

SHLIPAK, MICHAEL G, MD, MPH

PROFESSOR AND SENIOR RESEARCH SCIENTIST DEPARTMENT OF MEDICINE

UNIVERSITY OF CALIFORNIA SAN FRANCISCO VA MEDICAL CENTER

SAN FRANCISCO, CA 94121

GORDON, TERRY, PHD PROFESSOR

DEPARTMENT OF ENVIRONMENTAL MEDICINE SCHOOL OF MEDICINE

NEW YORK UNIVERSITY TUXEDO, NY 10987

SINGAL, AMIT, MD * HU, JIANZHONG, PHD

ASSOCIATE PROFESSOR ASSISTANT PROFESSOR

DIVISION OF LIVER AND DIGESTIVE DISEASES DEPARTMENT OF MEDICINE

SOUTHWESTERN MEDICAL CENTER DALLAS, TX 75201

TASEVSKA, NATASHA, MD, PHD * ASSISTANT PROFESSOR

SCHOOL OF NUTRITION AND HEALTH PROMOTION ARIZONA STATE UNIVERSITY

TEMPE, AZ 85281

DEPARTMENT OF GENETICS AND GENOMIC SCIENCES ICAHN SCHOOL OF MEDICINE AT MOUNT SINAI

NEW YORK, NY 10029

KEET, CORINNE, MD

ASSOCIATE PROFESSOR OF PEDIATRICS DVISION OF ALLERGY AND IMMUNOLOGY DEPARTMENT OF PEDIATRICS

JOHNS HOPKINS UNIVERSITY SCHOOL OF MEDICINE BLTIMORE, MD 21287

THOMSON, CYNTHIA A, PHD PROFESSOR

COLLEGE OF PUBLIC HEALTH ARIZONA CANCER CENTER UNIVERSITY OF ARIZONA TUCSON, AZ 85724

PONTARI, MICHEL, MD PROFESSOR

DEPARTMENT OF UROLOGY SCHOOL OF MEDICINE TEMPLE UNIVERSITY PHILADELPHIA, PA 19140

TRASANDE, LEONARDO, MD ASSOCIATE PROFESSOR DEPARTMENT OF PEDIATRICS LANGONE MEDICAL CENTER

NEW YORK UNIVERSITY SCHOOL OF MEDICINE NEW YORK, NY 10016

WOOD, ALEXIS CAROLINE, PHD * ASSISTANT PROFESSOR

CHILDREN'S NUTRITION RESEARCH CENTER PEDIATRICS BAYLOR OF COLLEGE OF MEDICINE

HOUSTON, TX 77030

MAIL REVIEWER(S) ASTOR, BRAD C, PHD ASSOCIATE PROFESSOR DEPARTMENT OF MEDICINE UNIVERSITY OF WISCONSIN MADISON, WI 53705

CHECKLEY, WILLIAM N, MD, PHD ASSISTANT PROFESSOR

DIVISION OF PULMONARY AND CRITICAL CARE SCHOOL OF MEDICINE

JOHNS HOPKINS UNIVERSITY BALTIMORE, MD 21205

RAMAKRISHNAN, USHA, PHD PROFESSOR AND VICE CHAIR ROLLINS SCHOOL OF PUBLIC HEALTH

HUBERT DEPARTMENT OF GLOBAL HEALTH ROLLINS SCHOOL OF PUBLIC HEALTH EMORY UNIVERSITY

ATLANTA, GA 30322

TUDOR-LOCKE, CATRINE E., PHD ASSOCIATE PROFESSOR

WALKING BEHAVIOR LABORATORY PENNINGTON BIOMEDICAL RESEARCH CENTER BATON ROUGE, LA 70808

VOLK, MICHAEL LUCAS, MD ASSOCIATE PROFESSOR

DIVISION OF GASTROENTEROLOGY AND NUTRITION SCHOOL OF MEDICINE

LOMA LINDA UNIVERSITY LOMA LINDA, CA 92350

SCIENTIFIC REVIEW OFFICER CHANETSA, FUNGAI, MPH, PHD SCIENTIFIC REVIEW OFFICER CENTER FOR SCIENTIFIC REVIEW NATIONAL INSTITUTES OF HEALTH BETHESDA, MD 20892

EXTRAMURAL SUPPORT ASSISTANT

KOSCINSKI, DANIEL

EXTRAMURAL SUPPORT ASSISTANT CENTER FOR SCIENTIFIC REVIEW NATIONAL INSTITUTES OF HEALTH BETHESDA, MD 20892

* Temporary Member. For grant applications, temporary members may participate in the entire meeting or may review only selected applications as needed.

Consultants are required to absent themselves from the room during the review of any application if their presence would constitute or appear to constitute a conflict of interest.
